# Molecular Insight into Gut Microbiota and Rheumatoid Arthritis

**DOI:** 10.3390/ijms17030431

**Published:** 2016-03-22

**Authors:** Xiaohao Wu, Bing He, Jin Liu, Hui Feng, Yinghui Ma, Defang Li, Baosheng Guo, Chao Liang, Lei Dang, Luyao Wang, Jing Tian, Hailong Zhu, Lianbo Xiao, Cheng Lu, Aiping Lu, Ge Zhang

**Affiliations:** 1 Institute for Advancing Translational Medicine in Bone & Joint Diseases, School of Chinese Medicine, Hong Kong Baptist University, Hong Kong SAR, China; wxho0606@163.com (X.W.); hebinghb@gmail.com (B.H.); liujin_hkbu@163.com (J.L.); lidefang@163.com (D.L.); borisguo@hkbu.edu.hk (B.G.); liangchao512@163.com (C.L.); danglei_hkbu@163.com (L.D.); luyaoben@126.com (L.W.); 2 Institute of Integrated Bioinformedicine and Translational Science, Hong Kong Baptist University, Hong Kong SAR, China; 3 Guanghua Integrative Hospital, Shanghai 200000, China; 13816671472@163.com (H.F.); mayinghui021@126.com (Y.M.); 4 Institute of Arthritis Research, Shanghai Academy of Chinese Medical Sciences, Shanghai 200000, China; 14441098@life.hkbu.edu.hk; 5 Research Group of Bone and Joint Diseases, HKBU Institute of Science & Technology, Haimen 226100, China; 6 Institute of Basic Research in Clinical Medicine, China Academy of Chinese Medical Sciences, Beijing 100000, China

**Keywords:** rheumatoid arthritis, gut microbiota, molecular mechanism

## Abstract

Rheumatoid arthritis (RA) is a systemic, inflammatory, and autoimmune disorder. Gut microbiota play an important role in the etiology of RA. With the considerable progress made in next-generation sequencing techniques, the identified gut microbiota difference between RA patients and healthy individuals provides an updated overview of the association between gut microbiota and RA. We reviewed the reported correlation and underlying molecular mechanisms among gut microbiota, the immune system, and RA. It has become known that gut microbiota contribute to the pathogenesis of RA via multiple molecular mechanisms. The progressive understanding of the dynamic interaction between gut microbiota and their host will help in establishing a highly individualized management for each RA patient, and achieve a better efficacy in clinical practice, or even discovering new drugs for RA.

## 1. Introduction

Rheumatoid arthritis (RA) is a chronic, inflammatory, and autoimmune disorder. The etiology of RA is multifactorial. From a vast selection of literature, it is clear that the pathogenesis of RA requires the interaction between genetic and environmental factors [1,2]. A low concordance rate of RA in monozygotic twins indicates the involvement of environmental factors in addition to the genetic risk factors [3]. It is well established that environmental factors, such as smoking and diet, have a close association with RA [4,5]. Previous reviews also highlighted the role of the microbiome, another environmental factor, in inflammatory arthritis and human rheumatic diseases [6]. Recently, emerging evidence indicates that the human gut microbiota may have a critical role in the pathogenesis of RA. Thus, in this article we summarized the current available data regarding to the correlation and underlying molecular mechanisms among gut microbiota, immune system, and RA.

## 2. The Human Gut Microbiota

As an important environmental factor to our body, the gut microbiota plays a major role in human health and disease. Indeed, it is in many ways an additional organ of the body [7]. The human gut alone harbors roughly three pounds of bacteria, whose collective genome encodes around three million different genes—100 times more than that of its human host [8]. It is estimated that 29% of all micro-organisms that live in and on the human body reside in the gut, which is the highest density recorded for any microbial habitat [9]. The gut microbiota co-develops with the host from birth, which contains a dynamic and vast array of micro-organisms that are helpful in digestion, nutrition uptake, energy harvest, vitamin synthesis, as well as providing important metabolic capabilities [10]. It is demonstrated that the presence of certain intestinal commensals can provide different signals for several aspects of intestinal development, including mucosal barrier fortification, angiogenesis, xenobiotic metabolism, and postnatal intestinal maturation [11].

During the period from birth to adulthood, gut microbiota co-evolves with the host. Symbiotic microbes may produce microbial compounds to inhibit the growth of pathogens and other transient invasive organisms, thereby protecting host from opportunistic invasion [12]. Fermentation, a metabolic process utilized by gut microbiota (e.g., *Clostridium*) which breaks down resistant starch or indigestible carbohydrates (dietary fiber) to short-chain fatty acids (SCFAs), is also critical to the physiological functions of host. SCFAs provide approximately 10% of the total dietary energy supply in humans, mainly including acetate, propionate, and butyrate. The gut microbiota could also shape the development and functions of host immune systems, triggering signals to instruct peripheral immune cells such as regulatory T (Treg) cell and T-helper (Th) cell differentiation. For example, it is recently demonstrated that gut microbiota-derived butyrate have the ability to induce the differentiation of colonic Treg cells [13]. *Bacteroides fragilis*, a normal symbiotic bacterium in the human gut, mediates the conversion of CD4+ T cells into Interleukin (IL)-10 producing Foxp3+ Treg cells through a specific molecule, polysaccharide A (PSA) [14]. In addition, this microbiome-derived molecule has been proven to promote immunologic tolerance via TLR2 signaling pathway directly on Foxp3(+) regulatory T cells [15]. Segmented filamentous bacterium (SFB), another symbiotic gut commensal, is found sufficient to induce the appearance and activation of Th17 cells in the lamina propria, which subsequently secrete pro-inflammatory cytokine (IL-17) and thereby enhance mucosal immune responses of the host [16]. On the other hand, the mucosal immune system is also a key factor to develop and maintain a healthy gut microbiota, which undergoes maturation alongside the gut microbiota continuously. By this interdependent functional relationship, the composition and functions of the gut microbiota play a critical role in regulating the Th17/Treg cells balance in the lamina propria and may thus influence host immune responses, tolerance, and susceptibility to autoimmune diseases.

A healthy gut microbiome is very important for the overall health of the host, which is often described as “homeostasis” [17]. However, the homeostasis of gut microbiota may be disturbed under certain pathological conditions. For instance, in genetically susceptible individuals, environmental factors (e.g., Smoke and dietary) can disturb the gut microbial communities (dysbiosis), causing dysregulations in host innate and adaptive immune systems, leading to the development of different diseases [18,19]. Recent studies have shown the involvement of gut microbiota in many autoimmune diseases, including autoimmune hepatitis [20], type 1 diabetes [21], multiple sclerosis [22], and spondyloarthritis [23]. Recently, increasing evidence indicates that the alterations in gut microbiota are also associated with RA development.

## 3. Alterations of Gut Microbiota in RA

Taking advantage of the culture-independent DNA sequencing technologies, it became possible to accurately identify the vast majority of microbiome living in our body cavities. While the fecal microbiome may not reflect all of the human microbiota, it offers a window into the overall gut microbiota. A previous study has shown that RA patients have significantly less *bifidobacteria* and microbiome of the *Bacteroides-Porphyromonas-Prevotella* group, *Bacteroides fragilis* subgroup, and *Eubacterium rectale--Clostridium coccoides* group in comparison to patients with non-inflammatory fibromyalgia [24]. In another study, researchers found a significant increase in the *Lactobacillus* communities in patients with early RA by comparing their fecal microbiome with those from healthy individuals, suggesting an alteration of *Lactobacillus* communities in early state of RA [25]. Scher *et al.*, identified a strong positive correlation between the *Prevotella* *copri* (*P. copri*) and new-onset untreated RA patients (NORA) [26]. *Prevotella* is found to be over-represented in NORA patients, and the relative abundance of *P. copri* in these patients negatively correlates with the presence of shared-epitope risk alleles. This report presents, for the first time, a close association between RA and a specific gut microbe. Intriguingly, the expansion of *P. copri* in RA patients could be suppressed to a similar state that was observed in the healthy subjects after RA treatment [26].

Using metagenomics shotgun sequencing, Zhang *et al.*, analyzed the fecal, dental, and salivary samples from a large cohort of RA patients and healthy controls, and found that there was a systematic alteration in the gut, dental, and saliva microbiota [27]. In concordance with the previous studies, results of fecal analysis showed that *Lactobacillus salivarius* was over-represented in RA individuals and was present in increased amounts in cases of very active RA, whereas a trend toward increased abundance of *P. copri* was observed as a function of RA duration in the first year. A large cluster including *Gordonibacter pamelaeae*, *Clostridium asparagiforme*, *Eggerthella lenta*, and *Lachnospiraceae bacterium* as well as small clusters containing strains such as *Lactobacillus* sp., *Bifidobacterium dentium* and *Ruminococcus lactaris* were enriched in the gut of RA patients. *Haemophilus* spp. was depleted in RA patients but enriched in healthy individuals. The observed gut microbial imbalance (dysbiosis) was also associated with clinical indices, such as the titers of immunoglobulin, autoantibodies, anticyclic citrullinated peptide (anti-CCP), and rheumatoid factor (RF). In addition, this gut dysbiosis can be partially reversed after treatment with disease modifying anti-rheumatic drugs (DMARDs) [27]. In addition, symbiotic relationships between the gut microorganisms have also been reported to be altered in patients with RA [28]. The fraction of *bifidobacteria*, *bacteroids*, and *lactopositive* *colibacteria* was reduced while the abundance of opportunistic *enterobacteria* and *staphylococci* was elevated. Meanwhile, the presence of opportunistic *Enterobacteriaceae* in urine and nasal mucosa further suggested the microbial translocation from the intestines. The authors concluded that changes in intestinal microflora and colonization by opportunistic bacteria may enhance the risk of developing co-morbid conditions in patients with RA [28].

Generally, the current evidence supports the notion that the alterations in gut microbiota distinguish RA patients from healthy individuals and are associated with the development and progression of RA (Table 1). However, due to the limited sample size, the above studies may not reflect the true alterations in the gut microbiota between RA patients and healthy individuals. More data are required to characterize the association between gut microbiota and RA. Nonetheless, it draws attention to the potential contribution of the gut microbiota to autoimmunity diseases such as RA and the relevant clinical manifestations.

## 4. Gut Microbiota Contribute to the Pathogenesis of RA

The intestinal tract comprises the largest mucosal surface area between the environment and the host [29]. The intestinal epithelial cell layer is continuously renewed and is a physical barrier that modulates both innate and adaptive immune responses to separate potential environmental threats such as toxins and pathogens from underlying tissues. It also allows the absorption of water and nutrients essential to life, as well as prevention of the microbial infiltration and subsequent inflammatory immune responses [30]. In recent years, notable advances in understanding the pathogenesis of RA have been achieved. One of the major concepts developed by multiple investigations is that the systemic autoantibodies, including RF and anti-citrullinated protein autoantibodies (ACPAs), are well detectable before the onset of arthritis and may originate from a site outside of the synovial joints even in the absence of synovitis [31]. While the precise etiology of RA remains unknown, emerging evidence supports the hypothesis that the local autoimmune processes leading to RA autoimmunity might originate from the mucosal immune system [32].

**Figure 1:**
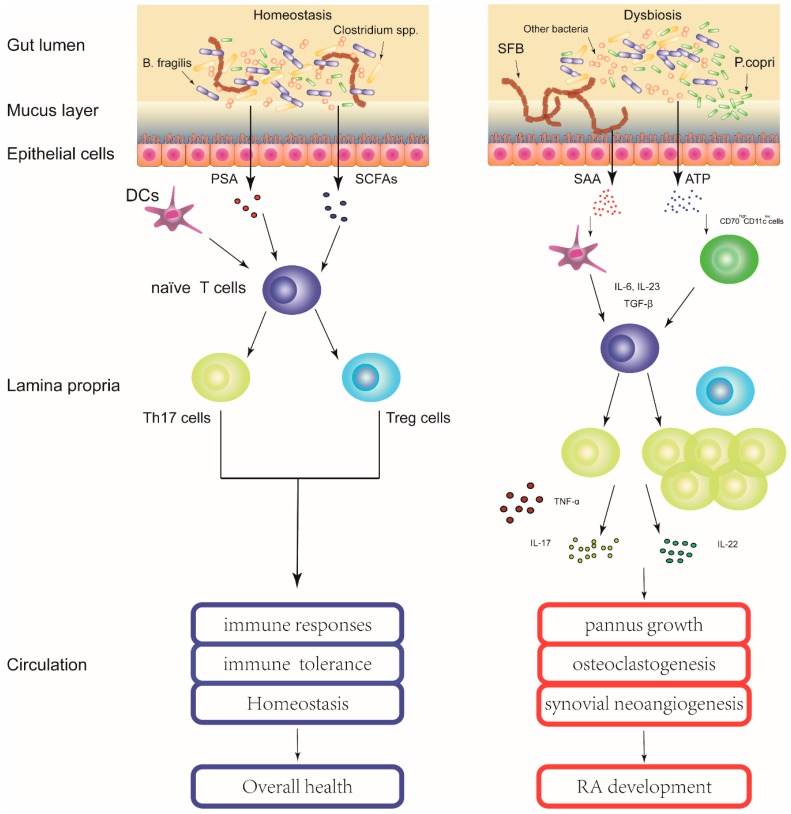
Gut microbiota contribute to the pathogenesis of RA. The healthy gut microbiota is in a homeostasis state that maintains integrity of the intestinal epithelial cell layer and has multiple symbiotic microbes to help in physiological functions. In genetically susceptible individuals, environmental factors can influence the gut microbiota causing changes in the types and abundance of microbiome (dysbiosis). The dysbiosis in gut microbiota, in association with genetic factors, may disrupt the innate and adaptive immune system and contribute to the development of RA via multiple molecular mechanisms. PSA, polysaccharide A; SCFAs, short-chain fatty acids; SAA, serum amyloid A; ATP, adenosine 5′-triphosphate; DCs, dendritic cells; IL, Interleukin; TGF-β, Transforming growth factor-beta; Treg cells, regulatory T cells; Th17 cells, T helper 17 cells.

Previous studies of germ-free and gnotobiotic animal models indicated that the presence of certain gut microbiome species may affect the development of autoimmune arthritis. While IL-1 receptor antagonist knock-out (Il1rn^–/–^) mice (spontaneously develop autoimmune arthritis) do not get arthritis under germ-free conditions, mono-colonization of *Lactobacillus bifidus* with Il1rn^–/–^ mice resulted in rapid onset of arthritis. The authors demonstrated that, in this model, the onset of arthritis was dependent on Toll-like receptor activation by *L. bifidus*. [33]. Another example comes from the K/BxN mouse model. Mono-colonization of single gut microbiome SFB with germ-free K/BxN mouse is sufficient to induce fully functional T_H_ 17 cells that produce pro-inflammatory cytokines IL-17 and drive the onset of arthritits [34]. In this inflammatory process, SFB upregulates the production of acute-phase isoforms of serum amyloid A (SAA) in the ileum, which can act on dendritic cells (DCs) from the small intestinal lamina propria to induce naïve CD4+ T cells to differentiate into Th17 cells [16]. Additionally, commensal bacteria have been shown to produce and secrete large amounts of adenosine 5′-triphosphate (ATP). It was found that commensal-bacteria-deriven ATP could activate a unique subset of lamina propria cells, CD70^high^CD11c^low^ cells. In response to ATP stimulation, the CD70^high^CD11c^low^ subset cells could express Th17-prone molecules, such as IL-6, IL-23p19 and transforming-growth-factor-β-activating integrin-ɑV and -β8, and then preferentially induce Th17 differentiation [35]. Thus, gut microbiota could induce Th17 cell differentiation via multiple pathways, which was consistent with the reported elevated circulating Th17 cells populations in patients with RA [36] (Figure 1). Th17 cells are a subset of T cells secreting their signature cytokine interleukin (IL)-17, as well as various other pro-inflammatory cytokines like IL-21, IL-22, granulocyte–macrophage colonystimulating factor (GM-CSF) and tumor necrosis factor ɑ (TNF-ɑ). These cells and cytokines have been implicated in the pathogenesis of RA considering their functional roles in mediating pannus growth, osteoclastogenesis, and synovial neoangiogenesis. For instance, *in vivo* and *in vitro* experiments have consistently shown that IL-17 and IL-22 could induce the RANKL expression in human synovial fibroblasts, leading to the loss of the RANKL/OPG balance and the subsequently enhanced osteoclastogenesis and bone erosion in autoimmune arthritis [37,38]. In addition, IL-17 could increase the production of vascular endothelial growth factor (VEGF) in rheumatoid fibroblast like synoviocytes (FLS), contributing to the angiogenesis in rheumatoid synovium [39]. Moreover, IL-17 could stimulate the expressions of various pro-inflammatory cytokines (e.g., IL-1β, TNF-α and IL-6) and matrix degrading enzymes (e.g., matrix metalloproteinase (MMP)-1, -2, -9, and -13) in whole synovial tissue, synovial fibroblasts, and cartilage, thus promoting inflammation, matrix turnover, and cartilage destruction during RA development [40,41].

Apart from the involvement of T cells, results from studies on human fecal microbiota also add clues for understanding the linkage between gut microbiota and RA. For example, the expansion of intestinal *P. copri* correlates with enhanced susceptibility to RA. Colonization of mice intestines with *P. copri* can enhance experimental dextran sulfate sodium-induced colitis and thus may have a pro-inflammatory potential in human arthritis [26]. In addition, the *P. copri* genome encodes phosphoadenosine phosphosulfate reductase, an oxidoreductase that participates in the production of thioredoxin. Thioredoxin has been widely implicated in the pathogenesis of RA and significantly increased concentrations of thioredoxin have been observed in both serum and synovial fluid of RA patients [42]. Although the direct mechanism has not yet been elucidated, this finding suggests that the overgrowth of *P. copri* may have a pathogenic role in the development of NORA. It also supports the hypothesis that normal intestinal microbiota and their degradation products may be involved in the development of autoimmune arthritis in genetically susceptible individuals, which was previously proposed by Toivanen in 2003 [43]. Moreover, the gut microbiota also have the capacity to influence autoimmune disease incidence in genetically predisposed animal models by altering sex-hormone levels [44]. Another *in vivo* study demonstrated that the gut microbiota, in association with HLA genes, determine the innate and adaptive immune system and contribute to the susceptibility to arthritis [45].

Taken together, the underlying mechanistic relationships among gut microbiota, immune system and RA are under intense investigations. Even though it is still challenging to define the exact role of gut microbiota in the etiology of RA, current evidence suggests that gut microbiota indeed contribute to the pathogenesis of RA via multiple potential molecular mechanisms. More mechanistic studies are needed to elucidate how gut microbiota contribute to the development of RA.

## 5. Perspective: Gut Microbiota as Potential Drug Targets for RA?

In 2008, a perspective review from *Nature Reviews Drug Discovery* pointed out that the gut microbiota is a potential new territory for drug targeting [46]. Recent studies have provided evidences to show that drugs may exert therapeutic effects through latent microbiota-mediated mechanisms [47,48,49]. The enrichment of beneficial gut microbiome and reduction of pathogen-like gut microbiome occurred before significant improvement of disease symptoms, suggesting that the rebalance of gut microbiota might contribute to the improvement of disease symptoms rather than a mere consequence after the symptoms have been alleviated [48]. Alterations of the gut microbiota composition in RA patients to some extent are associated with clinical efficacy, indicating that the gut microbiota may contain potential therapeutic targets for RA [27]. Animal experiments demonstrated the ability of gut microbiota in driving inflammation or the onset of autoimmune arthritis, such as *P. copri* and SFB. Thus, it is plausible to hypothesize that targeting the postulated pathogenic microbiome in the gut could contribute to clinical improvement in RA. Although there is a paucity of direct experimental data to validate this hypothesis, it is still supported by some circumstantial evidence. For example, medications with antimicrobial properties, such as minocycline and sulphasalazine, have been used as DMARDs in clinical practice according to the guidelines of the American College of Rheumatology. Sulphasalazine is proven to exert effect majorly by the active antimicrobial component sulphapyridine [50]. Certain antibiotics (e.g., Clarithromycin) can relieve the signs and symptoms of active RA [51] and show beneficial effect in those RA patients who are not responsive to or cannot tolerate DMARDs [52]. However, in most of these cases, treatment regimens were not designed to directly target a well-defined, specific gut microorganism or certain species of bacteria, as disease target. Thus, conclusions could be confounded by the unexpected drug effects on the gut microbiota. Collectively, the current medications for RA are insufficient to target the pathogen-like gut microbiome due to the lack of specificity.

In addition, it is also important to consider the unexpected effects of medications currently used in the treatment of RA on the ecology of the gut microbiota community. Evidence from mice models indicate that alterations of gut microbiota via use of antibiotics may aggravate experimental arthritis symptoms probably due to the partial depletion of natural gut flora [53]. Non-steroidal anti-inflammatory drugs (NSAIDs), which are frequently used in modifying chronic inflammatory diseases like RA, are found to be responsible for gut mucosal injury and enteropathy in RA patients. NSAIDs alter the composition of gut microbiota and thus may exhibit unexpected effects on host-microbiota homeostasis. Treatment with probiotics, particularly *Bifidobacterium*, *Lactobacillus*, and *Faecalibacteriaum prausnitzii*, shows protective effects in NSAIDs-treated animal models [54]. Intriguingly, supplementation of probiotic microbiome or microbiome-derived molecules with immune-modulating properties (e.g., PSA, SCFAs) may also exert immune regulatory effects by decreasing serum pro-inflammatory cytokines and increasing the serum level of anti-inflammatory cytokines [55,56,57]. Although inconsistent findings are shown in the published results, the sophisticated mechanisms remain to be understood as well, developing therapeutics to modulate gut microbiota may be a potential strategy for RA treatment.

Considered with the results from metabonomics and metagenomics, the question is how to define “dysbiotic” gut microbiota that may relate to the development of RA. The identified difference of gut microbiota between patients with RA and healthy individuals would provide an updated understanding to answer this question. Well-designed investigations should be carried out to characterize the gut microbiota and their contribution to the pathogenic process of RA. There might be a new strategy targeting or manipulating the gut microbiota to restore the homeostasis of gut ecology in the host, and eventually to achieve a therapeutic effect. Further, new drugs specifically targeting the gut microbiota dysbiosis in RA treatment are under requirement.

## 6. Conclusions

Treatment for RA has evolved from a strategy of providing symptomatic relief to implementation of therapeutic regimens that impact disease activity and to slow or arrest structural joint damage. Although current therapies for RA, such as biologics, have achieved high levels of responsiveness in clinical practice, there is still no known cure for RA. As shown in this review, current findings suggest that alterations in gut microbiota are associated with RA development. The dysbiosis in gut microbiota may play an important part in the pathogenic process of RA through multiple molecular mechanisms. For long term purpose of disease management, further research is required to characterize the gut “dysbiosis” and its contribution to the development and progression of RA. The progressive understanding of the dynamic interaction between gut microbiota and host will help in establishing a highly individualized management for each RA patient, even to discover new drugs for the treatment of RA and achieve a better efficacy in clinical practice.

## Figures and Tables

**Table 1:** Alterations of gut microbiota related with RA.

Studygroups	Sample Type	Technology Employed	Bacterial Taxa (↓low, ↑enriched)	Ref.
Early RA (51) *vs.* Fibromyalgia (50)	Stool	16S rRNA hybridization, and DNA-staining	↓*Bifidobacteria*, ↓*Bacteroides-Porphyromonas-Prevotella*, ↓*Bacteroides fragile*, ↓*Clostridium coccoides*	[24]
Early RA (15) *vs.* Healthy (15)	Stool	Quantitative real-time PCR	↑*Lactobacillus*	[25]
New-Onset RA (44) *vs.* Healthy (28)	Stool	16S rRNA gene and WGS sequencing	↑*Prevotella copri*, ↓*Bacteroidetes*	[26]
RA (30) *vs.*Healthy (30)	Stool	16S rRNA gene and WGS sequencing	↑*Enterococci*, ↑*Clostridia*, ↑*Colibacteria*, *↓Lactobacteria*	[27]
Treatment-naïve RA (94) *vs.* Healthy (97)	Stool, Dental, Saliva	Metagenomic shotgun sequencing	↑*Lactobacillus salivarius*, ↑*Gordonibacter pamelaeae*, ↑*Clostridium asparagiforme*, …, ↓*Haemophilus* spp.	[28]

## Abbreviations

RA	Rheumatoid Arthritis
SCFAs	short-chain fatty acids
Treg cell	regulatory T cell
Th cell	T helper cell
PSA	polysaccharide A
SFB	Segmented Filamentous Bacterium
IL	Interleukin
NORA	new-onset untreated RA
anti-CCP	autoantibodies anticyclic citrullinated peptide
DMARDs	disease modifying anti-rheumatic drugs
ACPAs	anti-citrullinated protein autoantibodies
SAA	serum amyloid A
ATP	adenosine 5′-triphosphate
GM-CSF	granulocyte–macrophage colonystimulating factor
TNF-α	tumor necrosis factor ɑ
VEGF	vascular endothelial growth factor
FLS	fibroblast like synoviocytes
MMP	matrix metalloproteinase
NSAIDs	non-steroidal anti-inflammatory drugs
